# Neglected synovial osteochondromatosis of the elbow: a rare case

**DOI:** 10.1186/1477-7819-11-233

**Published:** 2013-09-17

**Authors:** Silvio Giannetti, Attilio Santucci, Alessandro Patricola, Andrea Stancati, Vincenzo Di Sanzo

**Affiliations:** 1 Department of Orthopedic Surgery, Casa di Cura “Villa Stuart”, Via Trionfale 5952, Roma 00135, Italy; 2 Department of Surgical Sciences, “Sapienza” University, Viale Regina Elena 324, Rome 00185, Italy

**Keywords:** Synovial Chondromatosis, Elbow joint, Metaplastic disorder, Tumor, Open synovectomy

## Abstract

**Background:**

Synovial osteochondromatosis is a benign metaplastic proliferative disorder of the synovium characterised by the formation of multiple cartilaginous nodules in the synovium, many of which detach and become loose bodies. The disease is characteristically monoarticular, most commonly involving the knee. A site in the elbow was first reported in 1918 by Henderson, but any joint may be involved. Very few cases of synovial osteochondromatosis of the elbow have been reported in the literature. The presenting symptoms are usually diffuse discomfort in the affected joint and decreased range of motion with an accompanying gritty or locking sensation. The treatment of choice is excision of the synovium and removal of the loose bodies.

**Case presentation:**

We report a rare neglected case covering a 32-year period of a locally aggressive synovial osteochondromatosis of the elbow in a 47-year-old man. Clinical examination revealed a significant increase in size of the left elbow compared to the contralateral one. The simple radiographs and the computed tomography showed multiple rounded, calcified bodies widespread throughout the elbow joint. At surgery we removed and counted a total of 312 loose bodies, varying in size from a few millimeters to 3 cm. The evaluation at 6 months postoperatively showed marked reduction in the volume of the elbow, improvement of extension and flexion and an increase of the Mayo elbow performance score from 50 points before surgery to 80 points at 6 months postoperative.

**Conclusion:**

Synovial osteochondromatosis is an uncommon condition characterized by the formation of multiple nodules of hyaline cartilage within the sub-synovial connective tissue. The differential diagnosis includes chronic articular infection, osteoarthritis, pigmented villonodular synovitis, mono-articular inflammatory arthritis and periarticular neoplasms like synovial sarcoma. The treatment of choice is excision of the synovium and removal of the loose bodies. The prognosis is good, but recurrences may occur if the removal is incomplete.

## Background

Synovial osteochondromatosis (SOC) is a benign metaplastic proliferative disorder of the synovium characterised by the formation of multiple cartilagenous nodules in the synovium, many of which detach and become loose bodies [1-3].

The disease is characteristically monoarticular, most commonly involving the knee [4]. A site in the elbow was first reported in 1918 by Henderson [5], but any joint may be involved [6,7]. Very few cases of SOC of the elbow have been reported in the literature [8].

The presenting symptoms are usually diffuse discomfort in the affected joint and decreased range of motion with an accompanying gritty or locking sensation [9]. The treatment of choice is excision of the synovium and removal of the loose bodies [10].

We report a rare neglected case covering a 32-year period of a locally giant SOC of the elbow in a 47-year-old man.

## Case presentation

**Figure 1:**
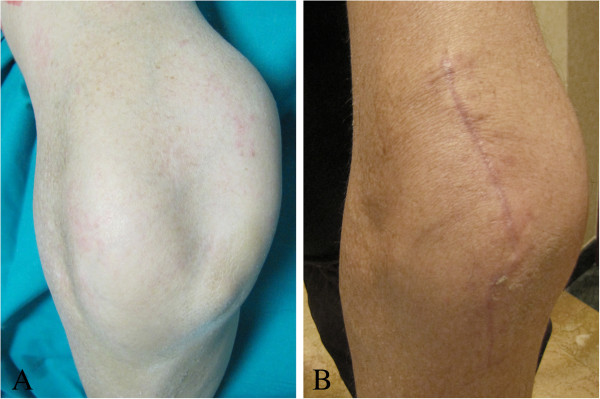
Aspect of the elbow (A,B) before and at 2 months postoperative.

A 47-year-old male, presenting a 32-year history of intermittent locking and loss of range of motion of the left elbow, with no recollection of associated trauma, was brought to our attention due to the considerable size of the elbow (Figure 1A), which had begun interfering with his job as a taxi driver and daily life. Clinical examination revealed a significant increase in size of the left elbow compared to the contralateral. The elbow lacked 40° of full extension and 85° of flexion and crepitation was noted when the elbow was brought from flexion to extension. Pronation and supination were substantially complete. Palpation of the elbow highlighted the presence of floaters in both the superficial and deep planes of the left elbow joint. No neurologic or vascular compression symptoms were observed. Routine laboratory data were normal. Other joints were normal.

**Figure 2:**
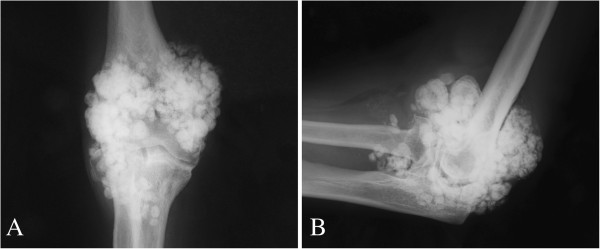
Anteroposterior (A) and lateral (Bx) radiographs of the left elbow show multiple rounded, calcified bodies widespread throughout the elbow joint.

**Figure 3:**
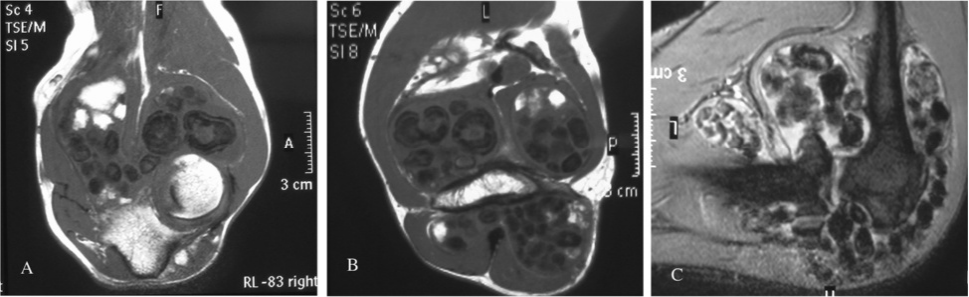
Magnetic Resonance (MR) images (A,B,C) show the distribution of the calcified bodies spread throughout the elbow joint.

The simple radiographs of the left elbow showed multiple rounded, calcified bodies widespread throughout the elbow joint (Figure 2A,B). Magnetic Resonance (MR) images showed the distribution of the calcified bodies spread throughout the elbow joint (Figure 3A,B,C). Diagnosis of synovial osteochondromatosis was made.

**Figure 4:**
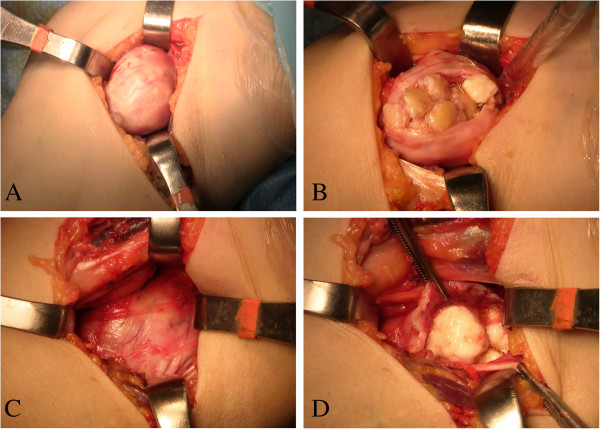
Anterior surgical access to the medial (A,B) and lateral (C,D) compartments of the elbow.

**Figure 5:**
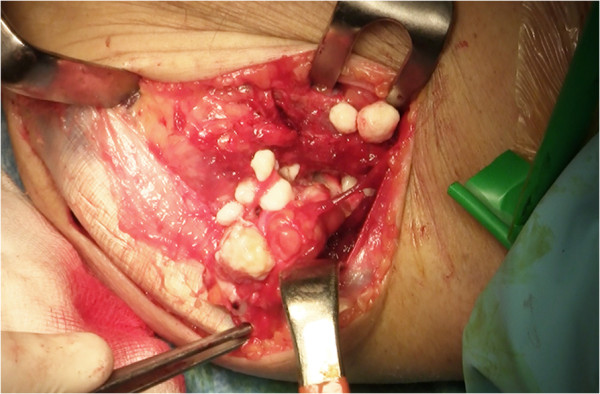
Posterior surgical access to the elbow.

**Figure 6:**
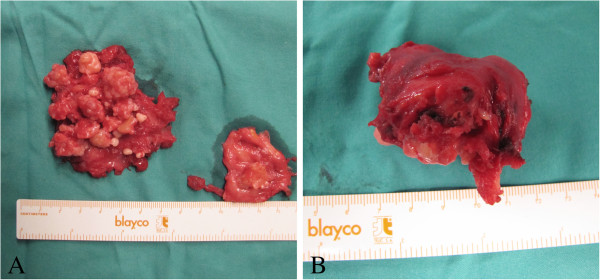
Multiple calcific bodies (A,B) were found adherent to the thickened synovium.

**Figure 7:**
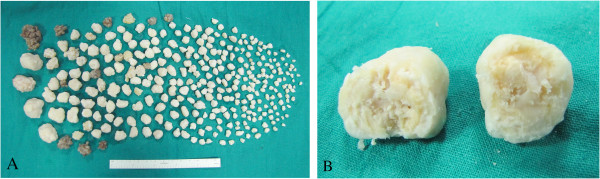
Macroscopic appearance of multiple cartilaginous nodules (A) and a calcified body dissected (B).

Open synovectomy was performed (by author A.S.) through anterior and posterior surgical access to the elbow. At surgery multiple calcific bodies were found adherent to the thickened synovium and within the joint space (Figures 4, 5 and 6). We removed and counted a total of 312 loose bodies, varying in size from a few millimeters to 3 cm (Figure 7). Histopathology confirmed the diagnosis.

**Figure 8:**
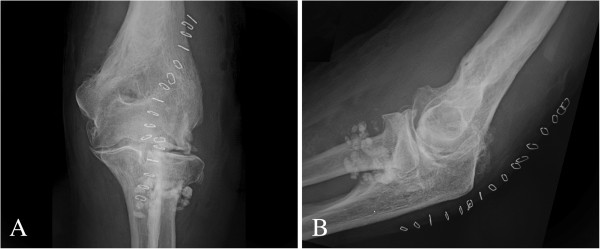
Anteroposterior (A) and lateral (B) postoperative radiographs of the left elbow.

**Figure 9:**
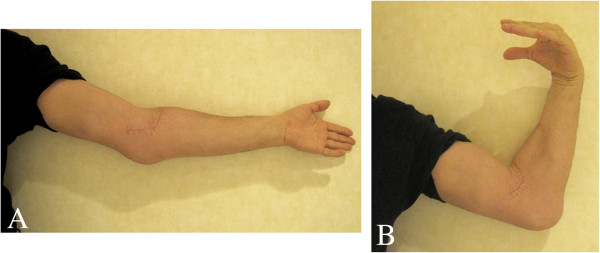
Range of motion of the left elbow (A,B) at 6 months postoperative.

Postoperatively, the elbow was supported in a sling for two weeks and then was mobilized progressively. The clinical and radiological evaluation at 6 months postoperatively showed marked reduction in the volume of the elbow, improvement of extension to 15° and flexion to 95°, no change in the preoperative prono-supination of the elbow, and no peripheral neurological deficits (Figures 8, 1 and 9).

The patient was assessed using the Mayo elbow performance score [11] before surgery and at 6 months postoperative, with an increase from 50 to 80 points.

## Conclusion

Synovial osteochondromatosis is an uncommon condition characterized by the formation of multiple nodules of hyaline cartilage within the sub-synovial connective tissue. The consensus regarding its pathogenesis is that it is due to hyperplastic metaplasia of the synovial connective tissue [12,13]. SOC is common in middle aged men. It is usually monoarticular and involves the knee in more than 50% of cases. The other common sites involved are hip, elbow, shoulder and ankle [13].

The more common symptoms associated with articular SOC include pain, swelling, loss of motion, and locking, and the most common physical signs consist of soft-tissue swelling, crepitation, palpable loose bodies, and limitation of motion [10]. The differential diagnosis includes chronic articular infection, osteoarthritis, pigmented villonodular synovitis, mono-articular inflammatory arthritis and periarticular neoplasms like synovial sarcoma [13].

The treatment of choice is excision of the synovium and removal of the loose bodies [10]. The purpose of the surgery, even in cases less overt, should aim not only to improve symptoms and function of the elbow but also prevent late degenerative joint disease. In preoperative planning, we preferred to consider the dual surgical access, anterior and posterior, for perimitral exposure of the elbow, rather than the medial and lateral surgical access. We believe that this choice has allowed a better control of the vascular and neuro structures and an easier radical synovectomy, especially in conditions like this, with significant distension of the elbow and subsequent probable dislocation of nerves and arteries.

The prognosis is good, but recurrences may occur if the removal is incomplete [13].

Despite the advanced state of the disease in our patient, the only symptoms and signs presented were related to the progressive increase in volume of the elbow, hard palpable loose bodies, locking of the elbow and grating during joint movement. There were no other symptoms or signs concerning significant pain, nerve palsy and bursitis. The surgical synovectomy and removal of floaters has helped improve the range of motion and function of the elbow.

## Consent

Written informed consent was obtained from the patient for publication of this Case report and any accompanying images. A copy of the written consent is available for review by the Series Editor of this journal.

## Abbreviations

SOC: Synovial osteochondromatosis; MR: Magnetic resonance.

## Competing interests

The authors declare that they have no conflict of interest and sources of financial support to the publication of this article.

## Authors’ contributions

1) SG, AS, AP, AS, VDS provided substantial contributions to conception and design, acquisition of data, or analysis and interpretation of data; 2) SG, AS, AP, AS, VDS provided drafting the article or revising it critically for important intellectual content; and 3) SG, AS, AP, AS, VDS provided final approval of the version to be published. All authors read and approved the final manuscript.
